# Growth and Development of *Helicoverpa armigera* (Lepidoptera: Noctuidae) Larvae Infected by *Heliothis virescens ascovirus* 3i (HvAV-3i)

**DOI:** 10.3389/fphys.2020.00093

**Published:** 2020-03-05

**Authors:** Gong Chen, Hang Liu, Bo-Cheng Mo, Jue Hu, Shuang-Qing Liu, Carlos Bustos-Segura, Jing Xue, Xing Wang

**Affiliations:** ^1^Hunan Provincial Key Laboratory for Biology and Control of Plant Diseases and Insect Pests, College of Plant Protection, Hunan Agricultural University, Changsha, China; ^2^College of Plant Protection, Hunan Agricultural University, Changsha, China; ^3^Institute of Biology, Faculty of Science, University of Neuchâtel, Neuchâtel, Switzerland

**Keywords:** *Heliothis virescens ascovirus* 3i, *Helicoverpa armigera*, pathogenic ability, mortality, insect virus

## Abstract

Although the cotton bollworm *Helicoverpa armigera* has traditionally been controlled by application of chemical pesticides, chemical control selects for resistance, pollutes the environment, and endangers human health. New methods for controlling *H. armigera* are therefore needed. *Heliothis virescens ascovirus* 3i (HvAV-3i) is a recently identified virus of the lepidopteran larvae. We tested the effects of HvAV-3i on *H. armigera* larvae following oral ingestion of HvAV-3i-containing hemolymph (about 1.0 × 10^10^ virus genome copies per larvae) and following injection of HvAV-3i-containing hemolymph by insertion of a needle. Following oral ingestion, first-instar to fifth-instar larvae grew and developed normally. Following needle injection, in contrast, the corrected mortality of third and fourth instars was 88.9 ± 2.1 and 93.7 ± 3.4%, respectively. Food intake was significantly lower for larvae injected with virus-containing hemolymph than with virus-free hemolymph. Larvae injected with virus-containing hemolymph had extended survival times and could not complete the pre-pupal stage. These results indicate that inoculation of HvAV-3i via needle injection, but not via oral ingestion, significantly reduced the growth and development of *H. armigera* larvae.

## Introduction

The cotton bollworm, *Helicoverpa armigera* Hübner (Lepidoptera: Noctuidae), is a worldwide crop pest that attacks over 200 species in nearly 20 families (Wang et al., 2009; Bird, 2017). Control of *H. armigera* has mostly depended on chemical insecticides and BT cotton (Guo, 1997; Ahmad et al., 1999; Wu and Guo, 2005). As a result of the continuous and widespread use of Bt cotton and long-term, large-scale, and indiscriminate chemical pesticide use, *H. armigera* has developed resistance to Bt cotton and many pesticides (Wakil et al., 2009, 2010; Liu et al., 2010; Zhang et al., 2012; Mironidis et al., 2013; Qayyum et al., 2015). To manage this pest, new methods of control, including biological control, are needed.

Ascoviruses are pathogens of insects that have the potential to provide biological control of *H. armigera*. Ascoviruses (*Ascoviridae*) were discovered in the larvae of *Helicoverpa zea* (Boddie) in the late 1970s and were named in the early 1980s (Adams et al., 1979; Hamm et al., 1986; Hudson and Carner, 1987). The genus *Ascovirus* contains five recognized species, including *Spodoptera frugiperda ascovirus* 1a, *Tricoplusiani ascovirus* 2a, *Heliothis virescens ascovirus* 3a (HvAV-3a), *Diadromus pulchellus* 4a, and *Tricoplusiani ascovirus* 6a (Bigot et al., 2011; Wei et al., 2014). Each ascovirus virion particle has a cyclic, super-helix double-stranded DNA genome (Federici, 1983; Asgari et al., 2017). Ascoviruses replicate intracellularly in their larval hosts and are highly pathogenic, causing failure to pupate in infected insect larvae. Ascoviruses typically induce cell rupture and the release of vesicles containing virus particles into the larval hemolymph (Federici et al., 1990; Asgari et al., 2017). Before they die, ascovirus-infected larvae exhibit weak muscle elasticity, decreased food intake, reduced weight gain, retarded growth, a yellow body color, and molting failure (Carner and Hudson, 1983; Federici and Govindarajan, 1990; Govindarajan and Federici, 1990). Ascoviruses are seldom transmitted via oral ingestion (Hamm et al., 1986; Federici and Govindarajan, 1990) but are often transmitted during oviposition by hymenopteran endoparasitoids (Bigot et al., 1997; Glynn Tillman et al., 2004; Stasiak et al., 2005). As a variant of HvAV-3a, the ascovirus HvAV-3i was first collected from infected *Spodoptera frugiperda* J.E. Smith (Lepidoptera: Noctuidae) larvae from south Florida, United States (Hamm et al., 1986) and was transmitted between noctuid larvae by braconid wasps (Stasiak et al., 2005). Although the effects of HvAV-3h on noctuid larvae have been studied (Li et al., 2013), the effects of HvAV-3i on *H. armigera* have not been studied.

In the current report, we describe the effects of HvAV-3i on larval instars of *H. armigera*, and we consider the potential of HvAV-3i as a biological control agent of this pest.

## Materials and Methods

### Insects and Viruses

A *H. armigera* population was established at the Virus Research Institute of Hunan Agricultural University. *H. armigera* larvae were reared on a pinto bean-based diet and were maintained in an incubator at 27 ± 1°C and with a relative humidity of 70% and a light:dark photoperiod of 14:10 h. Adults were supplied with a 10% honey solution. A laboratory stock of HvAV-3i was maintained by inoculating the third-instar larvae of *Spodoptera litura* Fabricius (Lepidoptera: Noctuidae) with *S. litura* hemolymph containing HvAV-3i; this was done by inserting an HvAV-3i-contaminated needle into a proleg of each larva. Hemolymph containing HvAV-3i was originally obtained from laboratory storage. Six days after inoculation, HvAV-3i-containing hemolymph was harvested by removing one proleg of each of the infected *S. litura* larvae. The virus-containing hemolymph was obtained by applying pressure to the larvae; the expressed hemolymph was collected in a 1-ml sterile vial and was used immediately or stored at −20°C.

### Effect of HvAV-3i Concentration on the Mortality of Needle-Inoculated *H. armigera* Larvae

The hemolymph of HvAV-3i-infected *S. litura* was serially diluted to obtain 0- to 10^–7^-fold dilutions with sterile water. Each of the eight dilutions was inoculated into the third-instar larvae (L3) of *H. armigera* by inserting a sterile fine needle (Brand: Braun; Specification: 0.3 mm × 8 mm) into a dilution and then inserting the tip of the needle (about 0.5 mm) into a proleg of an *H. armigera* larva; as a control, the larvae were inoculated with hemolymph from healthy *S. litura* larvae. Each combination of dilution and treatment (virus or mock inoculation) was represented by three replicates with 30 individuals per replicate. After inoculation, every individual was placed in a separate container with a diameter of 2 cm and a height of 2.5 cm. All larvae were fed with 27 mm^3^ of artificial diet (corn flour, soybean flour, yeast powder, agar, sorbic acid, sodium paraben, vitamins, cholesterol, tomato paste, etc.) (Burton, 1970) and kept at 27 ± 1°C with a relative humidity of 70% and a light:dark photoperiod of 14:10 h. We changed the fresh feed every 3 days. Each larva was observed daily for death and pupation, and the experiment was terminated after all larvae were dead or finished pupation.

### Viral Transmission *per os*

Before feeding separate groups of 90 first-instar (L1) and 90 second-instar (L2) larvae at a room temperature of 27°C, the surface of the diet was coated with HvAV-3i-containing hemolymph. After 48 h, when all the virus-containing diet had been consumed, the average food intake was used to calculate the mean virus dosage ingested per individual. The two separate larval groups were then given fresh diet. Mortality was assessed daily until all larvae were dead or finished pupation.

First-instar (L1) and second-instar (L2) larvae were placed in separate plastic containers with a diameter of 8 cm and a height of 8.5 cm (90 larvae per container). Each container contained 1 cm^3^ of artificial diet, the surface of which had been evenly coated with 100 μl of HvAV-3i-containing hemolymph (about 1.0 × 10^10^ genome copies per μl). The containers were kept at 27°C. After 48 h, when all the virus-containing diet had been consumed (the quantity of food ingested was used to calculate the quantity of virus ingested per larva), and then all the larvae were single-head fed with fresh diet in plastic containers with a diameter of 2 cm and a height of 2.5 cm. Controls were identical except that the diet was coated with hemolymph from healthy insects. Each treatment (L1 or L2 larvae that were fed hemolymph that contained or did not contain HvAV-3i) was represented by 90 replicate containers. Mortality was assessed daily until all larvae were dead or finished pupation.

Third-instar (L3), fourth-instar (L4), and fifth instar (L5) larvae were placed in separate plastic containers with a diameter of 2 cm and a height of 2.5 cm (a larvae per container). Each container contained 27 mm^3^ of artificial diet, the surface of which had been evenly coated with 1 μl of HvAV-3i-containing hemolymph (about 1.0 × 10^10^ genome copies per μl). The containers were kept at 27°C. After 48 h, when all the virus-containing diet had been consumed (the quantity of food ingested was used to calculate the quantity of virus ingested per larva), the larvae were then given fresh diet. Controls were identical except that the diet was coated with hemolymph from healthy insects. Each treatment (L3, L4, and L5 larvae that were fed hemolymph that contained or did not contain HvAV-3i) was represented by 90 replicate containers. Mortality was assessed daily until all larvae were dead or finished pupation.

### Viral Transmission by Needle Injection

L3 and L4 larvae were inoculated with virus (about 150–200 virions/cell) by pushing an HvAV-3i-contaminated needle into the left third proleg; L1 and L2 larvae were not included in this experiment because they were too small to inoculate with a needle. A total of 180 L3 larvae and 180 L4 larvae were selected for inoculation with hemolymph (0.16 ± 0.01 μl undiluted hemolymph per larva) by needle as described by Federici et al. (1990). Half of the L3 and L4 larvae were inoculated with virus-containing hemolymph, and the other half were inoculated with hemolymph from a healthy insect. The larvae were then placed in containers with a diameter of 8.5 cm and a height of 8 cm. One larva was placed with 27 mm^3^ of block feed in the container at 27°C. Death and pupation were recorded daily, survival curves were plotted, and mortality rates were calculated. Larval body weight and the quantity of diet ingested (the reduction after feeding) were determined daily with an electronic balance (Shimadzu AUY120) until the larva died or pupated. The loss of moisture from the diet by evaporation was calculated as previously described (Li et al., 2013). The daily food consumption and the total food intake were compared to the control group separately.

### Statistical Analyses

Adjusted mortality rate of treated groups was obtained using the one-way ANOVA procedure; before analysis, the data were determined to have a normal distribution. Larval body weight and food intake data were tested for a normal distribution and were examined with one-way analyses of variance (α = 0.05) using SPSS 22.0. Graphs were created using the SigmaPlot 12.5 software, and the larval mortality rates were calculated using the following formula: mortality rate = number of dead larvae/total number of larvae × 100%. The median survival times and 95% confidence intervals were obtained through the Kaplan–Meier method (SPSS). The log-rank test was used to determine treatment differences in survival times (*P* < 0.05), and survival curves were created with the GraphPad Prism 6 software.

## Results

### Mortality of Needle-Inoculated *H. armigera* as Affected by HvAV-3i Concentration

**FIGURE 1:**
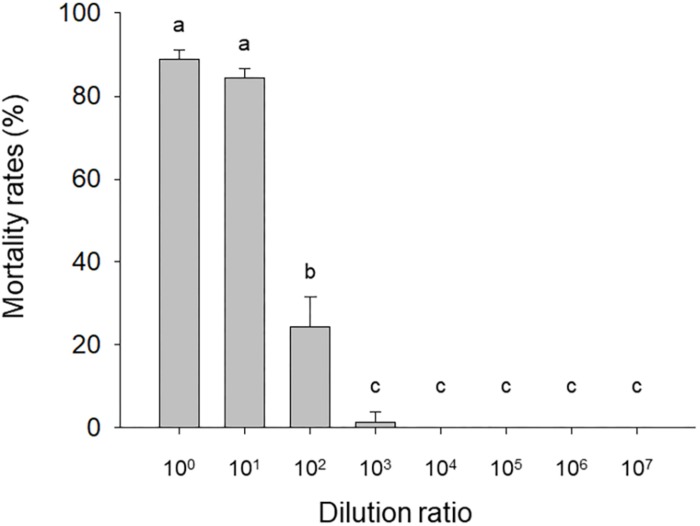
Mortality of needle-inoculated *Helicoverpa armigera* as affected by HvAV-3i concentration. Third-instar larvae of *H. armigera* were inoculated (via insertion of a needle into a proleg) with serially diluted hemolymph obtained from *Heliothis virescens ascovirus* 3i (HvAV-3i)-infected *Spodoptera litura* larvae (about 1 × 10^13^ genome copies per mL before dilution). Values are means ± SE of three replications, with 30 larvae per replication. Means with different letters are significantly different (*P* < 0.05, one-way ANOVA, LSD).

The mortality of needle-inoculated *H. armigera* larvae was significantly affected by HvAV-3i hemolymph dilution (*F*_7_,_16_ = 419.05, *P* < 0.0001). Mortality exceeded 80% with the undiluted and 10^1^-fold diluted virus-containing hemolymph but rapidly dropped with additional dilutions (Figure 1). Mortality for larvae inoculated with virus-free hemolymph was <12.5% regardless of the dilution.

### Mortality and Survival Time After *per os* Inoculation

Based on the quantity of hemolymph-coated diet ingested, each larva ingested about 1 × 10^10^ genome copies. The mortality of L1, L2, L3, L4, or L5 larvae observed among larvae that ingested virus-containing hemolymph or virus-free hemolymph was >90% of the larvae developed and pupated.

### Mortality and Survival of Needle-Inoculated L3 and L4 Larvae

**FIGURE 2:**
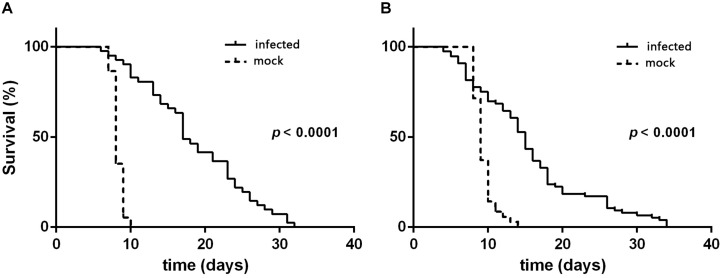
Survival curves of *H. armigera* larvae that were needle inoculated with virus-containing hemolymph (+HvAV-3i) or virus-free hemolymph (-HvAV-3i) (mock). **(A)** Third-instar larvae. **(B)** Fourth-instar larvae. Survival of the control larvae was considered to terminate when the larvae pupated.

According to the quantitative results of the virus, the amount of virus genome copies given to individual L3 to L4 was 1.56 × 10^9^. During the 32 days after inoculation, the mortality rate was 88.89 ± 2.14 and 93.65 ± 3.36% for L3 and L4 larvae that were needle inoculated with virus-containing hemolymph, respectively, and was 4.44 ± 1.11 and 11.10 ± 1.10% for L3 and L4 larvae that were needle inoculated with virus-free hemolymph, respectively. Mortality did not significantly differ between virus-inoculated L3 and L4 larvae (*F*_7_,_16_ = 0.34, *P* = 0.727). The median survival time (ST50) was significantly longer for virus-inoculated L3 and L4 larvae than for the mock-inoculated group larvae (Table 1). When plotted overtime, survival was also significantly greater for larvae inoculated with virus-containing hemolymph than for larvae inoculated with virus-free hemolymph (L3: χ^2^ = 62.64, *df* = 1, *P* < 0.0001, Figure 2A; L4: χ^2^ = 33.35, *df* = 1, *P* < 0.0001; Figure 2B). This difference in survival time was explained by the pupation rate, which was 99% for mock-inoculated larvae and 0% for virus-inoculated larvae.

**TABLE 1:** Survival time (days) of L3 and L4 larvae needle inoculated with virus-containing hemolymph or virus-free hemolymph.

Hemolymph treatment	L3		L4	
	ST_50_	95% CI	ST_50_	95% CI
With virus	17.0 ± 1.1 a	14.9–19.1	15.0 ± 0.7 a	13.7–16.3
Without virus	8.0 ± 0.2 b	7.7–8.3	9 ± 0.2 b	8.5–9.5

*ST50, median survival time in days; 95% CI, 95% confidence interval. Values are means ± SE of 90 replications. Larvae that pupated were recorded as not surviving (nearly all of the larvae inoculated with virus-free hemolymph pupated, but very few of the larvae inoculated with virus-containing hemolymph pupated). Means in a column followed by different letters are significantly different (*P* < 0.05).*

### Body Weight and Feeding of L3 and L4 Larvae That Were Needle-Inoculated With Virus-Containing or Virus-Free Hemolymph

**FIGURE 3:**
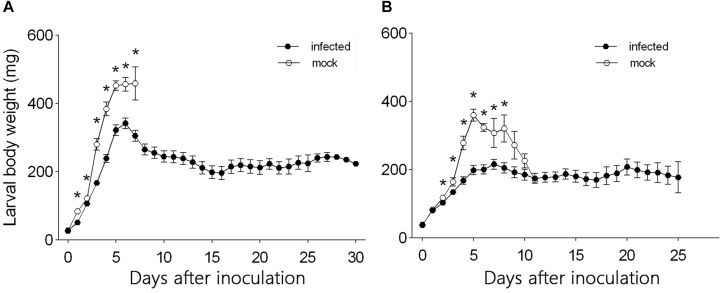
Daily body weight of **(A)** L3 larvae and **(B)** L4 larvae of *H. armigera* that were needle inoculated with virus-containing hemolymph (+HvAV-3i) or virus-free hemolymph (-HvAV-3i). Values are means ± SE. On each day, an asterisk indicates a significant difference between the +HvAV-3i and -HvAV-3i (control) groups (*P* < 0.05, one-way ANOVA).

From day 1 to day 7 after inoculation, the body weight was significantly smaller for L3 larvae that were needle inoculated with virus-containing hemolymph than for L3 larvae that were needle inoculated with virus-free hemolymph (*F*_1_,_124_ = 60.45, *P* < 0.0001 for day 1; *F*_1_,_124_ = 7.26, *P* = 0.008 for day 2; *F*_1_,_124_ = 74.26, *P* < 0.0001 for day 3; *F*_1_,_124_ = 43.837, *P* < 0.0001 for day 4; *F*_1_,_124_ = 18.29, *P* < 0.0001 for day 5; *F*_1_,_124_ = 11.81, *P* = 0.001 for day 6; *F*_1_,_124_ = 6.35, *P* = 0.014 for day 7) (Figure 3A). The body weight of L3 larvae that were inoculated with virus-containing hemolymph increased rapidly from day 0 to day 6 after inoculation but gradually decreased thereafter. The body weight of L3 larvae that were inoculated with virus-free hemolymph increased to day 7, at which time 92.5% of the larvae pupated.

Body weight did not differ significantly between L4 larvae inoculated with virus-containing hemolymph and L4 larvae inoculated with virus-free hemolymph on days 0 and 1 and on days 9, 10, and 11 after inoculation (*F*_1_,_117_ = 1.26, *P* = 0.26 for day 0; *F*_1_,_117_ = 0.15, *P* = 0.70 for day 1; *F*_1_,_117_ = 1.70, *P* = 0.20 for day 9; *F*_1_,_117_ = 0.39, *P* = 0.54 for day 10; *F*_1_,_117_ = 0.01, *P* = 0.94 for day 11) (Figure 3B). Body weight was significantly smaller for L4 larvae inoculated with virus-containing hemolymph than for L4 larvae inoculated with virus-free hemolymph on days 2–8 after inoculation (*F*_1_,_117_ = 4.13, *P* = 0.04 for day 2; *F*_1_,_117_ = 5.02, *P* = 0.03 for day 3; *F*_1_,_117_ = 17.37, *P* < 0.0001 for day 4; *F*_1_,_117_ = 46.80, *P* < 0.0001 for day 5; *F*_1_,_117_ = 33.42, *P* < 0.0001 for day 6; *F*_1_,_117_ = 4.59, *P* < 0.04 for day 7; *F*_1_,_117_ = 6.90, *P* = 0.01 for day 8) (Figure 3B). The body weight of the L4 larvae that were inoculated with virus-containing hemolymph increased during the first 5 days after inoculation and then remained relatively stable until the end of the experiment.

**FIGURE 4:**
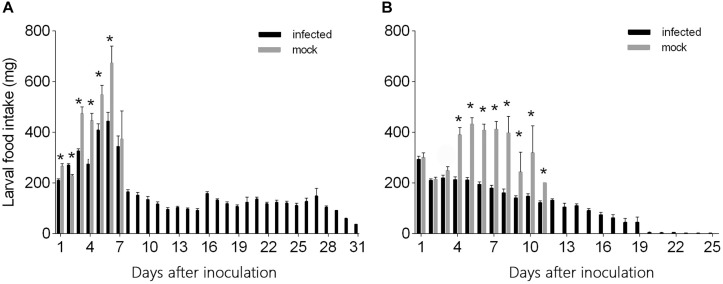
Daily food intake by **(A)** L3 and **(B)** L4 larvae of *H. armigera* that were needle inoculated with virus-containing hemolymph or virus-free hemolymph (mock). Values are means ± SE. On each day, an asterisk indicates a significant difference between the inoculated group and the mock-infected group (*P* < 0.05, one-way ANOVA).

L3 larvae inoculated with virus-containing hemolymph ingested less food than L3 larvae inoculated with virus-free hemolymph at days 1–6 after needle inoculation (*F*_1_,_124_ = 29.26, *P* < 0.0001 for day 1; *F*_1_,_124_ = 17.87, *P* < 0.0001 for day 2; *F*_1_,_124_ = 49.10, *P* < 0.0001 for day 3; *F*_1_,_124_ = 22.05, *P* < 0.0001 for day 4; *F*_1_,_124_ = 7.32, *P* = 0.008 for day 5; *F*_1_,_124_ = 8.42, *P* < 0.005 for day 6) (Figure 4A). From day 7 to day 31, however, food ingestion did not differ between the two groups. The quantity of food ingested by L3 larvae that were inoculated with virus-free hemolymph increased from day 1 to day 6 but then declined to zero as the larvae entered the pre-pupal stage (Figure 4A). The quantity of food ingested by L3 larvae that were inoculated with virus-containing hemolymph also increased from day 1 to day 6 but then declined and remained relatively low and stable from day 8 to day 31, i.e., the virus-inoculated L3 larvae survived and continued to ingest small quantities of food until day 31 (Figure 4A).

The quantity of food ingested did not significantly differ for L4 larvae inoculated with virus-containing hemolymph vs. L4 larvae inoculated with virus-free hemolymph on days 1–3 after needle inoculation (*F*_1_,_117_ = 0.29, *P* = 0.59 for day 1; *F*_1_,_117_ = 0.13, *P* = 0.72 for day 2; *F*_1_,_117_ = 2.156, *P* = 0.145 for day 3) (Figure 4B). From day 4 to day 12, L4 larvae inoculated with virus-containing hemolymph ingested less food than L4 larvae inoculated with virus-free hemolymph (*F*_1_,_117_ = 45.79, *P* < 0.0001 for day 4; *F*_1_,_117_ = 91.80, *P* < 0.0001 for day 5; *F*_1_,_117_ = 99.37, *P* < 0.0001 for day 6; *F*_1_,_117_ = 57.22, *P* < 0.0001 for day 7; *F*_1_,_117_ = 22.99, *P* < 0.0001 for day 8; *F*_1_,_117_ = 8.17, *P* = 0.007 for day 9; *F*_1_,_117_ = 15.73, *P* < 0.0001 for day 10; *F*_1_,_117_ = 4.26, *P* = 0.048 for day 11) (Figure 4B). From day 4 to day 8, food ingestion by the L4 larvae that were not inoculated with virus was relatively stable; these L4 larvae entered the pre-pupal stage on day 6, and their food ingestion dropped on day 9 and ceased on day 12. The quantity of food ingested by virus-inoculated L4 larvae gradually decreased from day 1 to day 31 (Figure 4B).

### Total Food Ingested by L3 and L4 Larvae That Were Needle Inoculated With Virus-Containing or Virus-Free Hemolymph

The total quantity of food ingested per larva was significantly greater for virus-inoculated L3 larvae than for virus-free L3 larvae but did not significantly differ between virus-inoculated L4 larvae and virus-free L4 larvae (L3: *F*_1_,_5_ = 10.20, *df* = 1, *P* = 0.002; L4: *F*_1_,_5_ = 2.29, *df* = 1, *P* = 0.133) (Table 2).

**TABLE 2:** Total food ingested by L3 and L4 larvae that were needle inoculated with virus-containing hemolymph or virus-free hemolymph.

Hemolymph treatment	mg of food ingested per L3 larva	mg of food ingested per L4 larva
With virus	2568 ± 85a	1829 ± 76a
Without virus	2109 ± 83b	2025 ± 89a

*Values are means ± SE of 90 replications. Means in a column followed by different letters are significantly different (*P* < 0.05).*

## Discussion

In the current study, we analyzed the effects of HvAV-3i on the growth and development of *H. armigera.* Compared to baculoviruses and nuclear polyhedrosis viruses, ascoviruses have a low toxicity and cause only low mortality in assays involving transmission via oral ingestion (Govindarajan and Federici, 1990; Caballero et al., 1992; Senthil-Nathan and Kalaivani, 2005, 2006). We previously reported that only a few early instar larvae of *S. exigua* were infected by HvAV-3h following oral ingestion (Hu et al., 2016). The current study was consistent with that earlier study in that no mortality occurred when L1–L5 larvae of *H. armigera* were inoculated with HvAV-3i via oral ingestion. Although HvAV-3i and HvAV-3h were isolated in different host generations and on different continents (Hamm et al., 1985; Huang et al., 2012; Chen et al., 2018, 2019), they are similar in being poorly transmitted or not transmitted at all via oral ingestion.

Hamm et al. (1986) found that ascoviruses are mainly spread in the field by the oviposition behavior of parasitic wasps. In attempt to mimic transmission via wasp oviposition, we used a needle to inject HvAV-3i into *H. armigera* larvae. In contrast to oral ingestion, pin inoculation resulted in mortality rates that exceeded 80% (Figure 1), and those larvae that survived were unable to enter the pre-pupal stage. Although the mortality rates caused by HvAV-3i inoculation of *H. armigera* larvae were high, they were not as high as those caused by HvAV-3h inoculation of *S. exigua* larvae (Hu et al., 2016). The survival time was also longer for HvAV-3i-inoculated *H. armigera* larvae than for HvAV-3h-inoculated *S. exigua* larvae (Hu et al., 2016). As a consequence, the pool of HvAV-3i inoculum may persist longer than the pool of HvAV-3h inoculum in the field. Together, the current results indicate that HvAV-3i may be useful for the biological control of *H. armigera*.

Given that ascoviruses are transmitted by parasitic wasps, the simultaneous application of parasitic wasps and ascoviruses should be considered as a biological control strategy (Stasiak et al., 2005). During oviposition, the parasitoid’s ovipositor can be contaminated with virions circulating in the hemolymph of the caterpillar (Asgari et al., 2017). The ascovirus can then be transmitted when the parasitoid wasp oviposits in another host (Chen et al., 2018, 2019). The effects of HvAV-3i on *H. armigera* and the interactions between HvAV-3i and parasitoid wasps should be investigated in the field.

## Conclusion

The current results indicate that the HvAV-3i virus cannot infect *H. armigera* larvae by oral ingestion but can infect *H. armigera* larvae when the virus is needle inoculated into L3 and L4 larvae. Needle inoculation of *H. armigera* larvae led to a high infection rate, rapid pathogenicity, sharply reduced feeding, greatly reduced gains in body weight, and the prolonged duration of larval stages. The results therefore indicate that HvAV-3i has a potential as a biological control agent of *H. armigera* and that HvAV-3i-infected *H. armigera* may be useful for increasing and maintaining the pools of HvAV-3i inoculum in the field.

## Data Availability Statement

The datasets generated for this study are available on request to the corresponding author.

## Author Contributions

JH and XW contributed to the data curation. HL, JH, and B-CM contributed to the formal analysis. GC, HL, S-QL, JX, and JH contributed to the investigation. GC and XW contributed to the methodology, the project administration, and the validation. GC, HL, and JH contributed to the writing of the original draft. GC, XW, and CB-S contributed to the reviewing and editing aspect of the writing for the manuscript.

## Conflict of Interest

The authors declare that the research was conducted in the absence of any commercial or financial relationships that could be construed as a potential conflict of interest.
